# Vitamin D deficiency increases with age and adiposity in Emirati children and adolescents irrespective of type 1 diabetes mellitus: a case control study

**DOI:** 10.1186/s12902-023-01405-3

**Published:** 2023-07-14

**Authors:** Maria Majeed, Mohsin Siddiqui, Nader Lessan

**Affiliations:** Imperial College London Diabetes Centre, Khaleej Al Arabi Street, PO Box 48338, Abu Dhabi, UAE

**Keywords:** 25(OH)D, Adiposity, Emirati Children and Adolescents, Type 1 Diabetes, Normoglycaemia, Paediatric population, Obesity

## Abstract

**Background:**

Association of vitamin D (25(OH)D) deficiency with obesity and diabetes has been well-established in paediatric and adult populations. This study aims to report the association of 25(OH)D deficiency with body composition and prevalence of 25(OH)D deficiency in Emirati children and adolescents, who attended a diabetes centre in the United Arab Emirates.

**Methods:**

Using Abu Dhabi Diabetes and Obesity Study cohort, type 1 diabetes (T1D) and normoglycaemic (NG) participants between 4–19 years of age were selected. WHO criteria were used to define 25(OH)D cut-offs: deficient (< 30 nmol/L), insufficient (30-50 nmol/L) and sufficient (> 50 nmol/L). Based on CDC recommendations, BMI percentile was categorised as underweight, normal weight, overweight and obesity.

**Results:**

After age and sex matching, 148 T1D cases and 296 NG controls were identified. 25(OH)D deficiency was observed in 22.3% (*n* = 33) T1D and 40.5% (*n* = 120) NG participants. 25(OH)D levels were lower in adolescents (15 – 19 years) than children (4 – 7 years) in both T1D and NG groups (*p* = 0.018 vs *p* < 0.001). Females were more likely to be 25(OH)D deficient in both groups. Children and adolescents with BMI ≥ 95^th^ percentile were more likely to be 25(OH)D deficient than those with normal weight (OR: 2.69; 95% CI: 1.56, 4.64). Adiposity measures and 25(OH)D levels correlated negatively in both groups (T1D *p* < 0.01, NG *p* < 0.001).

**Conclusion:**

Vitamin D 25(OH)D deficiency is notably prevalent in Emirati children and adolescents despite adequate sunlight throughout the year. The prevalence was lower in those with T1D which may be indicative of treatment compliance in this population. This study also confirms important negative association of serum 25(OH)D levels with body mass and obesity in this population.

## Background

Vitamin D deficiency is a public health concern worldwide with a high global prevalence. Serum 25-hydroxy vitamin D (25(OH)D) levels reliably reflect total vitamin D stores and are frequently used in routine clinical care and research. The role of severe 25(OH)D deficiency (typically levels < 30 nmol/l) in the development of rickets and osteomalacia is well established [1]. There is growing evidence that low levels of 25(OH)D are associated with diabetes, cardiovascular disease, autoimmune conditions and neurocognitive disorders [2–5]. Impaired pancreatic beta-cell function and insulin resistance have been linked to 25(OH)D deficiency [6].

In the United Arab Emirates (UAE), 25(OH)D deficiency is endemic despite adequate sunlight throughout the year [7]. 25(OH)D deficiency (< 50 nmol/L) was observed in 72% of 12,346 participating adults attending ambulatory health care centres in Abu Dhabi [8]. Using a 25(OH)D cut-off ≤ 37.5 nmol/L, 25(OH)D deficiency was reported in 19.7% of adolescents aged 15–19 years [9]. Another study in Emirati school-going female adolescents detected 25(OH)D deficiency (< 27.5 nmol/L) in 78.8% of participants [10].

25(OH)D deficiency has been linked to the risk of developing type 1 diabetes (T1D) and many have proposed its protective and therapeutic role in reducing that risk [11, 12]. An inverse association between vitamin D supplementation during pregnancy and the presence of islet antibodies in the offspring has been reported [13]. The presence of vitamin D receptors (VDRs) on immune cells and the association of VDR gene polymorphism with susceptibility to T1D further suggest its immunomodulatory role in development of T1D [14, 15].

Coinciding with the high prevalence rates of 25(OH)D deficiency, there has been a dramatic rise in obesity prevalence in recent decades. Studies have reported an association between low serum 25(OH)D levels and obesity parameters such as body mass index (BMI), fat mass and waist-to-hip ratio [16, 17]. A meta-analysis of 25 observational studies showed an inverse association of serum 25(OH)D levels with percentage fat mass (PFM). However, it didn’t demonstrate an effect of vitamin D supplementation on PFM [18]. The lower serum 25(OH)D concentration in obesity is attributed to its sequestration in adipose tissue which reduces its bioavailability [19]. Other suggested mechanisms include volumetric dilution, impaired 25-hydroxylation of vitamin D in the liver and adipose tissue, decreased cutaneous synthesis, reduced sun exposure and impaired expression of vitamin D receptors (VDRs) [20–22].

In the UAE, the relationship between 25(OH)D levels and obesity has only been studied in obese adults with type 2 diabetes. Sadiya et al. found an inverse correlation between serum 25(OH)D levels and BMI and fat mass index in this population [23]. However, the relationship of serum 25(OH)D levels and adiposity in the paediatric age group with and without T1D remains unexplored in this region. Thus, this study aimed to determine; (i) the prevalence of 25(OH)D deficiency and insufficiency in normoglycaemic (NG) children and adolescents and those with T1D attending a large diabetes centre (ii) factors associated with 25(OH)D deficiency in this age group, and (iii) the association between serum 25(OH)D levels and adiposity measures.

## Methods

### Study design, setting and participants

We conducted an age and sex-matched case–control study in those with T1D and NG controls in the paediatric age group (4 – 19 years), who were originally recruited for the Abu Dhabi Diabetes and Obesity study (ADOS). The ADOS study protocol has been described in detail previously (clinical trial registration number: NCT01843959). In brief, the ADOS study was designed to investigate the factors associated with diabetes and obesity in the Emirati population. The ADOS study recruited participants aging 4–80 years between March 2013 to April 2017 at Imperial College London Diabetes Centre (ICLDC) by convenience sampling. ADOS participants were recruited from various BMI classes and glycaemic status to achieve a balanced distribution across groups. ICLDC is a large outpatient facility in the Emirate of Abu Dhabi, specialising in diabetes and endocrinology, and incorporating services in primary care, cardiology, ophthalmology, nephrology and occupational health. Patients from across UAE attend ICLDC and are predominantly of Arab Emirati origin. Venous blood samples were obtained after taking informed consent from the parents or guardian of the participating children. The study was approved by ICLDC institutional Research Ethics Committee (IREC008) and followed the principles of Declaration of Helsinki, 1996.

### Anthropometric and biochemical measurements

In this study, we classified participants into two age groups; children aged 4 to < 10 years, and adolescents aged ≥ 10 to 19 years. Anthropometric measurements were carried out by trained nurses. Weight and height were measured to the nearest 100 g and 0.5 cm respectively. Body composition comprising of fat mass, fat percentage, and fat free mass was assessed by bioelectrical impedance analysis (Tanita®, Tokyo, Japan). This method analyses body composition using the rate at which electrical current conducts through the body. The adipose tissue offers greater resistance to electrical flow than other tissues. The device uses a proprietary formula to estimate fat mass, fat free mass, percentage body fat and body water [24]. Blood samples were collected for vitamin D (serum 25(OH)D), lipids, and HbA1c. Serum 25(OH)D was measured using Elecsys total II kit for Cobas platform (Roche Diagnostics, Indianapolis, Indiana). Inter- and intra- assay variabilities for serum 25(OH)D assay were 10.7% and 4.6%, respectively. Information on demographics and medical history including age at diagnosis of diabetes, pre-existing medical conditions, and prescribed medications was extracted from electronic medical records. Prescribed vitamin D supplements included vitamin D2 (oral ergocalciferol 50,000 IU or 300,000 IU injections) and vitamin D3 (oral cholecalciferol 1,000 IU or 400 IU/ml drops).

NG participants were defined as those with HbA1c lower than 5.7% or fasting plasma glucose < 5.6 mmol/, as per ADA guidelines [25]. These participants were otherwise healthy and did not have any known pre-existing conditions. Participants with a known diagnosis of T1D as defined by the presence of insulin islet antibodies and hyperglycaemia were included in the study [25]. The World Health Organization (WHO) and Global Consensus recommendations were used to define 25(OH)D levels as deficient (< 30 nmol/L), insufficient (30-50 nmol/L) or sufficient (> 50 nmol/L) [26, 27]. BMI was categorized according to Centre for Disease Control recommendations: underweight (< 5^th^ percentile), normal weight (5^th^—< 85^th^ percentile), overweight (85^th^—< 95^th^ percentile) and obese (≥ 95^th^ percentile) [28].

### Selection of cases and controls

We selected 736 children and adolescents with measured serum 25(OH)D for this study. Participants with T1D were chosen as cases. The NG controls were identified using Mahalanobis distance-based neighbourhood method for age and sex matching in a 2:1 ratio. Exact matching method was used for sex, and the nearest Mahalanobis distance for age ranged from 0 to 2.34 with a mean (SD) of 0.11(0.39) in 296 controls.

### Statistical analysis

Data are presented as numbers (percentages) for categorical variables, and median (interquartile range) for continuous variables. The Shapiro–wilk test and graphical analysis of histogram plots were used to test the normality of data. Wilcoxon rank-sum (Mann–Whitney) and Pearson's chi-squared tests were carried out to compare characteristics between cases and controls. Spearman’s correlation was used to determine the relation between 25(OH)D levels and adiposity measures. Conditional and unconditional logistic regression were used to estimate odds ratio for 25(OH)D deficiency (< 30 nmol/L). Results from age and sex adjusted unconditional multivariate logistic regression and conditional logistic regression were similar, with narrower confidence intervals in the unconditional model. Thus, only odds ratios from unconditional multivariate regression are presented. In the first model, age and sex adjusted odds ratios were calculated to minimize the confounding potentially introduced by matching [29]. Variables were included in the final multivariate logistic regression (stepwise method) while scrutinizing clinically relevant predictors only. Due to multicollinearity, only BMI categories as adiposity index were included in the final multivariate model. In the multivariate logistic regression model, diabetes status, vitamin D medications, BMI groups, age and sex were mutually adjusted, and model fit was assessed by a goodness of fit Hosmer–Lemeshow test. Statistical significance was assessed at the level of *p* < 0.05. Data showed non-parametric distribution and hence median and interquartile range (IQR) are reported for continuous variables. All statistical analyses were performed using Stata version 15 (StataCorp LLC, Texas, USA).

## Results

### Study population characteristics and prevalence of 25(OH)D deficiency

**Fig. 1:**
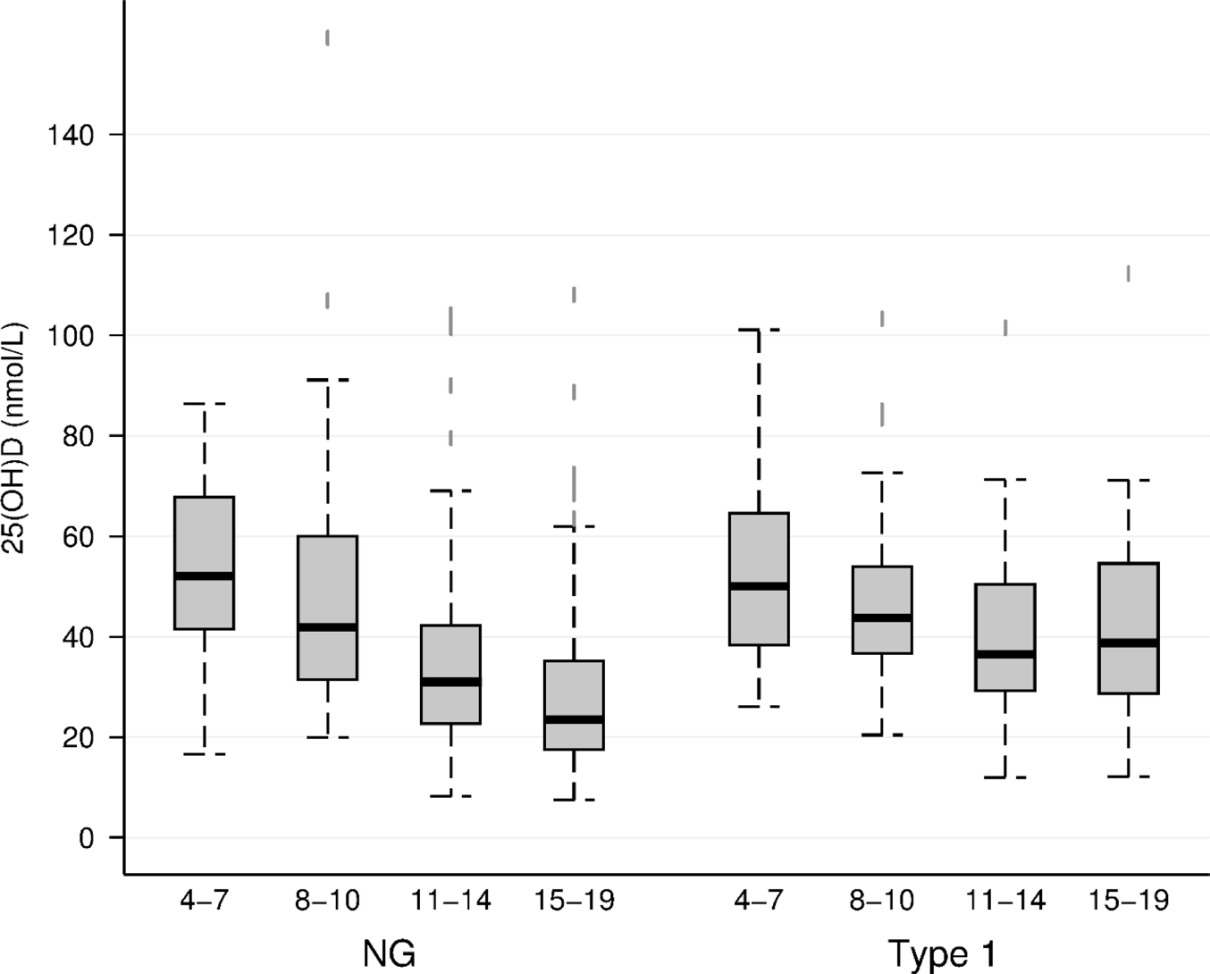
Relationship between 25(OH)D and age (years) in all subjects. Variation in 25(OH)D levels across age groups stratified by diabetes status. Tukey plot shows median, interquartile range (IQR) and IQR ± 1.5 * IQR. Pairwise comparisons were significant in NG individuals in all age groups except between 4–7 vs 8–10 years. In T1D individuals, pairwise comparisons were only statistically significant between 4–7 and 11–14 years. Multiple comaprisons were assesed using Kruskal-Wallis test followed by Dunn's test with Bonferroni adjustment

Baseline characteristics of 148 participants with T1D and 296 age and sex matched NG controls are shown in Table 1. 90% participants were of Emirati origin. The prevalence of 25(OH)D deficiency (< 30 nmol/L) was 22.3% (*n* = 33) in T1D and 40.5% (*n* = 120) in NG group. Serum 25(OH)D levels were higher in T1D than in NG group [median (IQR), 39.5(30.6, 54.3) vs 34.9 (23.5, 50.4), *p* = 0.0018]. Compared to NG group, a greater proportion of T1D participants were on vitamin D supplements (72.0% vs 82.4%, *p* = 0.015). BMI percentile across groups was comparable, with no significant difference (*p* = 0.42) (Table 1). Serum 25(OH)D levels decreased with increasing age in both groups, from 4–7 years to 15–19 years [T1D, *p* = 0.018; NG, *p* < 0.001] (Fig. 1).

**Table 1:** Baseline characteristics of T1D, and age and sex matched NG children and adolescents (age: 4–19 years)

Characteristics	NG	T1D	*P* value
N	296	148	
Age (years)	11.9 (10.1, 14.6)	12.5 (10.1, 14.9)	0.41
Adolescent (10-19 years)	225 (76.0%)	113 (76.4%)	0.94
Female	173 (58.4%)	82 (55.4%)	0.54
BMI Percentile	70.5 (20.5, 94.5)	63.5 (31.0, 88.0)	0.42
Vitamin D (nmol/L)	34.9 (23.6, 50.5)	39.5 (30.6, 54.4)	0.0018
Vitamin D cut-offs			
Sufficient (> 50 nmol/L)	75 (25.3%)	47 (31.8%)	< 0.001
Insufficient (30-50 nmol/L)	101 (34.1%)	68 (45.9%)	
Deficient(< 30 nmol/L)	120 (40.5%)	33 (22.3%)	
Vitamin D Medications	213 (72.0%)	122 (82.4%)	0.0156
HbA1c %	5.2 (5.0, 5.4)	9.5 (8.1, 10.8)	< 0.001
BP Systolic (mmHg)	107.0 (100.0, 117.0)	107.0 (98.5, 116.0)	0.40
BP Diastolic (mmHg)	61.0 (57.0, 67.0)	63.0 (57.0, 68.0)	0.13

Characteristics between T1D, and age and sex matched NG children and adolescents were compared, using Wilcoxon rank-sum test and Pearson’s chi-square test. Data are presented as median (IQR) for continuous variables and number (%) for categorical variables

Adolescents had a much higher prevalence of 25(OH)D deficiency than children in both NG and T1D groups [NG: 40.5% vs 14.1%, *p* < 0.0001; T1D:26.5% vs 8.5%, *p* = 0.026] (Table 2). A greater proportion of females were 25(OH)D deficient in both groups [NG: 52.6% vs 23.6%, *p* < 0.0001; T1D: 31.7% vs 10.6%, *p* = 0.0020]. After adjusting for age and sex, T1D participants were less likely to have 25(OH)D deficiency [Odds Ratio (OR): 0.39 (95% CI: 0.24, 0.62)] than the NG (Table 2). Children and adolescents with obesity (BMI ≥ 95^th^ percentile) had a greater risk of 25(OH)D deficiency [OR: 2.69; (95% CI: 1.56, 4.64)] than normal weight participants (BMI 5^th^—< 85^th^ percentile). In a mutually adjusted logistic regression model, T1D, obesity and vitamin D remained significant; T1D individuals were less likely to have 25(OH)D deficiency while those who were obese or on vitamin D supplementation were more likely to be 25(OH)D deficient (Table 2).

**Table 2:** Prevalence and association of 25(OH)D deficiency (< 30 nmol/L) in T1D and NG children and adolescents (*n* = 444)

	Prevalence of 25(OH)D deficiency (< 30 nmol/L)		Multiple Logistic Regression			
	Cases (T1D)	Controls (NG)	Age and Sex Adjusted	*P*-value^a^	Mutually Adjusted OR [aOR]	*P*-value*
Diabetes Status						
Normoglycaemia		120 (40.5%)	Ref			
Type 1	33 (22.3%)		0.39 (0.24, 0.62)	0.000	0.39 (0.24, 0.65)	0.000
Vitamin D Medication						
No	4 (15.4%)	17 (20.5%)	Ref			
Yes	29 (23.7%)	103 (48.4%)	2.08 (1.19, 3.63)	0.009	2.14 (1.20, 3.81)	0.010
BMI Group						
Normal Weight	18 (18.7%)	60 (38.5%)	Ref			
Underweight	2 (22.2%)	7 (22.6%)	0.77 (0.33, 1.79)	0.549	0.73 (0.31, 1.74)	0.482
Overweight	8 (29.6%)	13 (37.1%)	0.92 (0.49, 1.72)	0.801	0.90 (0.47, 1.72)	0.750
Obesity	5 (31.2%)	40 (54.0%)	2.69 (1.56, 4.64)	0.000	2.12 (1.21, 3.71)	0.008
Age (years)						
Children (4—< 10)	3 (8.5%)	10 (14.1%)	Ref			
Adolescent (10–19)	30 (26.5%)	110 (48.9%)	-		4.00 (2.06, 7.77)	0.000
Sex						
Male	7 (10.6%)	29 (23.6%)	Ref			
Female	26 (31.7%)	91 (52.6%)	-		3.19 (1.97, 5.16)	0.000

NG Normoglycaemic controls, T1D Type 1 Diabetes

^a^Age and sex adjusted, unconditional logistic regression

^*^ Mutually adjusted logistic regression, model fit was assessed by a goodness of fit Hosmer–Lemeshow test (*p* = 0.815)

### Body composition

**Fig. 2:**
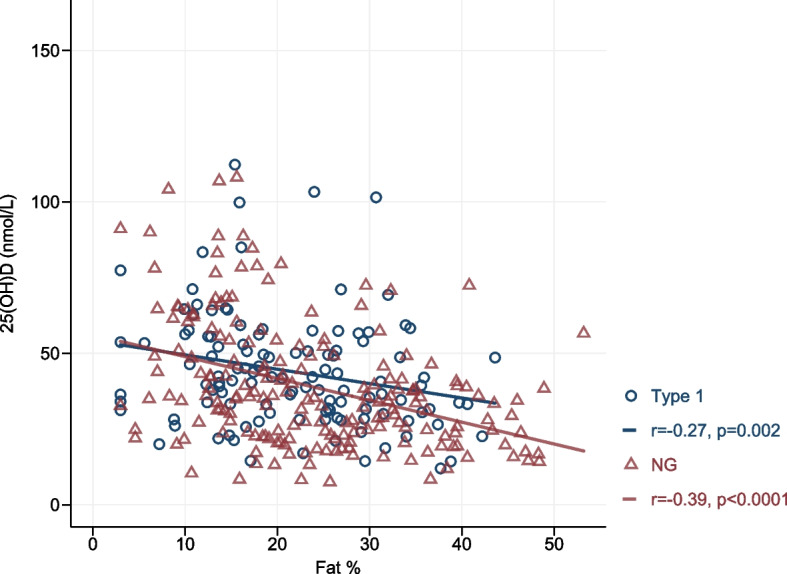
Spearman’s correlation between 25(OH)D and fat % in type 1 diabetes (*n* = 118) and NG group (*n* = 191)

Body composition using bioelectrical impedance analysis was available in 308 children and adolescents (117 in T1D and 191 in NG respectively). Fat mass was similar in both T1D and NG groups. Although a negative association was observed between fat mass and 25(OH)D levels in both groups (T1D *p* < 0.01, NG, *p* < 0.001) (Table 3), the association was much stronger in the NG group (Fig. 2). Similarly, fat mass, percentage fat mass and BMI percentile were higher across categories with decreasing 25(OH)D levels (Table 4).

**Table 3:** Correlation of 25(OH)D levels with adiposity measures in NG and T1D groups

	**NG**			**T1D**		
Parameters	NG	r_s_	*P*-value	T1D	r_s_	*P*-value
N						
BMI Percentile	70.5 (20.5, 94.5)	-0.259	0.0000	63.5 (31.0, 88.0)	-0.281	0.0005
Fat (%)	22.4 (14.8, 32.3)	-0.390	0.0000	21.4 (14.0, 29.1)	-0.272	0.0029
Fat Mass (kg)	9.2 (4.4, 17.4)	-0.438	0.0000	9.6 (5.3, 15.9)	-0.302	0.0009
Impedance	605.9 (548.7, 690.9)	0.237	0.0009	605.3 (554.2, 669.4)	0.199	0.0309

Fat %, Fat mass (kg) and impedance *N* = 191 in NG group and *N* = 117 in T1D group. NG Normoglycaemic controls, T1D Type 1 Diabetes

Values indicate median (IQR). r_s_ Spearman’s rank correlation coefficient

**Table 4:** Adiposity measures across 25(OH)D cut-offs as: sufficient (> 50 nmol/L), insufficient (30-50 nmol/L) and deficient (< 30 nmol/L)

NG				
Factor	**Sufficient (> 50 nmol/L)**	**Insufficient (30-50 nmol/L)**	**Deficient(< 30 nmol/L)**	*p*-value
N	46	69	76	
Fat %	14.7 (10.9, 19.9)	21.6 (14.9, 32.5)	27.4 (20.2, 36.5)	0.0001
Fat Mass	4.5 (3.0, 7.7)	8.3 (4.2, 17.4)	13.8 (8.2, 23.6)	0.0001
BMI Percentile	32.5 (5.0, 63.0)	75.0 (16.0, 95.0)	77.0 (28.0, 95.5)	0.0003
Impedance	650.5 (590.2, 703.5)	619.5 (553.8, 678.6)	578.5 (519.0, 642.0)	0.0101
T1D				
N	38	54	25	
Fat %	16.1 (11.9, 26.4)	22.4 (15.1, 29.6)	26.3 (16.6, 31.7)	0.021
Fat Mass	6.0 (3.3, 11.6)	10.4 (6.0, 15.9)	13.6 (6.6, 18.8)	0.005
BMI Percentile	41.5 (17.0, 69.0)	71.5 (51.0, 88.0)	76.0 (49.0, 92.0)	0.007
Impedance	625.1 (583.2, 706.5)	597.0 (533.1, 669.4)	593.1 (544.8, 642.8)	0.027

Comparison was carried out across 25(OH)D categories using Kruskal–Wallis test with Bonferroni adjustment. Total sample is restricted to number of patients undergoing bioelectrical impedance analysis. Median (IQR) are presented. NG normoglycaemic controls, T1D type 1 diabetes

## Discussion

Our study highlights the high prevalence of 25(OH)D deficiency among children and adolescents in the UAE and describes important associations between serum 25(OH)D and adiposity measures. Adolescents are at a higher risk of deficiency than children, consistent with other reports from the Middle East and Europe in people with or without diabetes [30–32]. Since vitamin D supplements are routinely recommended in children in the UAE, this could partly explain the higher, albeit insufficient serum 25(OH)D levels in this age group [33]. Additionally, females were more at risk of 25(OH)D deficiency, likely due to conservative clothing worn by women in the UAE, which can impede ultraviolet light absorption and vitamin D synthesis [34, 35]. Similar trends in vitamin D levels have been observed in other regions of the world where modest clothing is culturally practised [36–38]. Furthermore, female sex is consistently identified as a risk factor for hypovitaminosis D in both Western and developing countries, although the exact mechanism remains unclear [39].

Contrary to other case–control studies, the prevalence of 25(OH)D deficiency (≤ 30 nmol/L) was higher in NG controls (40.5%) control participants than those with T1D [40–42]. The higher concentration of serum 25(OH)D in T1D in our study is possibly due to more vitamin D prescriptions and better compliance. Another possible explanation could be that the T1D participants are more likely to get tested during their routine diabetes care appointments and subsequently offered supplementation We further evaluated this in a sub-analysis in those without vitamin D prescription which showed no difference in serum 25(OH)D between T1D (*n* = 83) and NG (*n* = 26) groups (56.9 nmol/l (42.2, 64.6) vs 55.3 nmol/l (33.7, 68.4), *p* = 0.903).

In our analysis, 25(OH)D deficiency was significantly higher in participants with obesity than in those of normal weight, even after adjusting for age, sex, diabetes and vitamin D supplementation. Additionally, serum 25(OH)D levels negatively correlated with multiple measures of adiposity in bioimpedance analysis in both healthy and T1D groups, which agrees with reports from China [43], Canada [44] and the United States [45]. However, the data on these associations in children and adolescents from the Middle East is sparse. In 477 healthy Iranian children aged 9 to 18 years, the fat mass index showed an inverse association with serum 25(OH)D concentration [16]. Another study in 4183 healthy Saudi school children reported a strong inverse association of serum 25(OH)D levels with adiposity indices [46]. This inverse association between adiposity measures and 25(OH)D in the paediatric age group with T1D has also been noted in other studies from Iran and Poland [47, 48].

Obesity is highly prevalent in the UAE and is being increasingly diagnosed in younger age groups [49, 50]. Although the rapid increase in prevalence in the Middle East is attributed to urbanisation and lifestyle changes, the pathophysiology of obesity is more complex. Studies have consistently demonstrated a predisposition to weight gain in susceptible individuals when exposed to environmental triggers [51]. The role of vitamin D deficiency as a contributing factor or consequence of obesity has not yet been established. Similarly, it is unclear whether vitamin D deficiency influences the development and progression of T1D. VDRs are expressed in pancreatic islets and islets appear to respond to locally produced 1,25 (OH)_2_D_3_, leading to the suggestion that adequate vitamin D levels are essential for optimal islet cell function [52]. While clinical studies have not consistently shown the benefits of vitamin D supplementation in reducing the risk of obesity or T1D [53], testing and supplementation in children and adolescents who are obese or overweight would not be unreasonable owing to potential beneficial effects on bone health, glycaemia and the immune system. Our study underscores the need for attention to vitamin D deficiency and adiposity in the paediatric age group with or without diabetes. We recommend that further interventional trials with vitamin D supplements are required to assess their effects on T1D and adiposity in this population.

The key strength of our study lies in the prospective collection of data and a relatively large sample size. The participants were well matched using the Mahalanobis distance method to minimise counterfactual bias. Furthermore, conditional and unconditional logistic regression models were compared, and model-fit was assessed.

However, our study has some limitations. The participants were recruited among those who were attending a large diabetes centre and were therefore not randomly selected. This may introduce bias, as many had diabetes, higher BMI percentiles or both. Moreover, a higher proportion of NG participants (25%) in the BMI ≥ 95^th^ percentile category was included than in the T1D group (10.8%) which could have caused confounding bias when comparing the two groups. Body composition was assessed using bioelectrical impedance analysis, which is comparatively less precise than other methods such as computed tomography (CT) and Dual-energy X-ray absorptiometry (DEXA) scan; however, it is not usually feasible to examine adiposity using these techniques in larger cohorts.

## Conclusion

Vitamin D deficiency is alarmingly prevalent among Emirati children and adolescents irrespective of diabetes status. We have found significant associations between lower 25(OH)D levels and obesity, specifically with body fat mass. Early intervention in this cohort is imperative to prevent potential long-term consequences to bone health, and possibly glycaemic control.

## Data Availability

The data that support the findings of this study are available on request from the corresponding author.

## Abbreviations

25(OH)D: 25-Hydroxy vitamin D
T1D: Type 1 Diabetes
NG: Normoglycaemic
UAE: United Arab Emirates
ICLDC: Imperial College London Diabetes Centre
ADA: American Diabetes Association
WHO: World Health Organisation
IQR: Interquartile range
BMI: Body Mass Index
HbA1c: Glycated Haemoglobin
